# 3D Graphene-Nitrogen Doped Carbon Nanotubes Network Modified Electrode as Sensing Materials for the Determination of Urapidil

**DOI:** 10.3390/ma11020322

**Published:** 2018-02-23

**Authors:** Yanju Wu, Anxing Zhou, Huimin Yang, Fei Wang, Kui Lu

**Affiliations:** 1 School of Material and Chemistry Engineering, Henan University of Engineering, Zhengzhou 450007, China; yjwu2008@163.com (Y.W.); AnxingZ_2014@163.com (A.Z.); HuiMY2018@163.com (H.Y.); 2 School of Chemical Engineering and Food Science, Zhengzhou Institute of Technology, Zhengzhou 450044, China

**Keywords:** 3D graphene, graphene-nitrogen doped carbon nanotubes, pulse potentiostatic method, electrochemical sensor, urapidil

## Abstract

In this work, a three dimensional (3D) graphene-nitrogen doped carbon nanotubes (G-NCNTs) network was successfully fabricated on the surface of a glassy carbon (GC) electrode using the pulse potential method (PPM) in a graphene oxide-nitrogen doped carbon nanotubes (GO-NCNTs) dispersion. The morphological and characteristics of GO-NCNTs and G-NCNTs nanocomposites were investigated by atomic force microscopy (AFM), scanning electron microscopy (SEM), UV-vis spectroscopy, Raman spectroscopy, and electrochemical experiments. The 3DG-NCNTs network was applied as a new voltammetric material for the fabrication of an electrochemical platform for determination of urapidil. Systematic electrochemical tests demonstrate that the 3DG-NCNTs network modified GC electrode can effectively increase the response to the oxidation of urapidil. Under the optimum conditions, the electrochemical response was linear with urapidil concentrations in the range of 1.0 × 10^−8^~2.0 × 10^−6^ mol·L^−1^, while a low detection limit of 5.0 × 10^−9^ mol·L^−1^ was obtained for urapidil. Moreover, the proposed sensing platform exhibited good results for sensitivity, reproducibility, selectivity, and stability, which makes it very suitable for use as an ideal inexpensive and rapid analytical method applicable for complex drug matrices.

## 1. Introduction

**Scheme 1:**
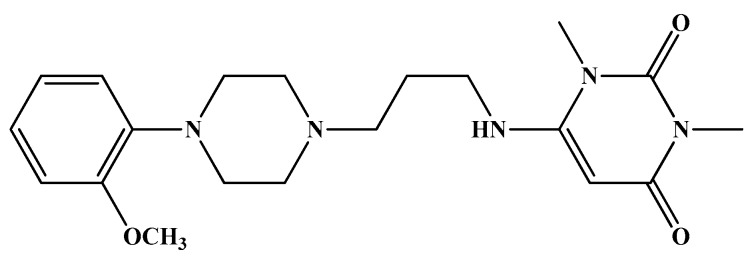
Chemical structure of urapidil.

Urapidil is a phenyl piperazine substituted uracil derivative and its structure is shown in Scheme 1. Because of its characteristic to induce blood vessel dilation with few serious side effects, it is very suitable to treat severe hypertension and hypertensive crisis for the elderly [1,2]. Now it has been applied as a preferential anti-hypertensive drug in many countries. Therefore, the determination of urapidil is necessary for drug safety. UV-vis and flow-injection chemiluminescence [3,4] was first employed, but it suffered from low detection limit and narrow linear range. Recently, several analytical methods, such as high performance liquid chromatography (HPLC) [5], liquid chromatography-mass spectrometry (LC-MS) [6,7], capillary electrophoresis (CE) [8], and atomic absorption spectrometery (AAS) [9], have been also proposed for determination of urapidil. Although these methods have some advantages of accuracy and sensitivity, they require tedious pretreatment procedures, or complicated pre-separation processes, at high cost. In contrast, electrochemical methods with respect to their advantages such as simplicity, sensitivity, accuracy, and ease of on-site applications, have received considerable attention. As to the determination of urapidil, different electrochemical methods have been reported [10,11]. Despite the progress made, it is very interesting and important to develop a rapid, simple, sensitive, and reliable analytical method for urapidil.

Graphene (as a two-dimensional (2D) carbon material) has been an attractive candidate for numerous applications including electronic devices [12], energy conversion and storage devices [13], sensors and biosensors [14] due to its excellent electrochemical stability, large specific surface area, and high electrical conductivity [15,16]. However, the aggregation or restacking of graphene sheets that inevitably occurs due to the strong π-π interactions results in the loss of effective surface area [17]. To overcome this issue, it is very important to design and fabricate three-dimensional (3D) graphene sheets with porous structures. These 3D graphene (3DG) networks have an extraordinary surface area, allow target analyte to access the individual graphene sheets more easily, and create continuous ion transport pathways due to their unique porous structure, which in turn decreases the mass transport resistance of the electrode. Recently, various methods have been proposed for fabricating 3D graphene including the template method [18], chemical reduction method [19], chemical vapor deposition (CVD) method [20], and the electrochemical reduction of graphene oxide (GO) sheets method [21]. Among these methods, electrochemical reduction is a green, facile, and low cost approach for preparing electroactive materials. Moreover, it is the most convenient and effective way for electrode surface modification since it can directly deposit reduced graphene sheets on the surface of electrodes. On the other hand, various electrochemical platforms based on carbon nanotubes (CNTs) have also been widely used in past years [22,23,24]. Besides, recent research confirmed that nitrogen-doped CNTs (NCNTs) exhibit better conductivity, as well as higher rate capability and wettability than that of ordinary CNTs [25,26,27].

In this work, we present a simple yet effective way to fabricate the 3D graphene-nitrogen doped carbon nanotubes (3DG-NCNTs) network modified glassy carbon (GC) electrode using the pulse potential method (PPM). Scanning electron microscopy (SEM) images and electrochemical experiments confirmed that G-NCNTs nanocomposite interpenetrated the network of 3D structure and gave excellent electrochemical performance. The 3DG-NCNTs network was applied as a new voltammetric material for the fabrication of an electrochemical platform for determination of urapidil. Systematic electrochemical tests demonstrated that the 3DG-NCNTs network modified GC electrode can effectively increase response to the oxidation of urapidil. Under the optimum conditions, the electrochemical response was linear with urapidil concentrations in the range of 1.0 × 10^−8^~2.0 × 10^−6^ mol·L^−1^. The proposed sensing platform exhibited good sensitivity, reproducibility, selectivity, and stability, which makes it very suitable for use as an ideal inexpensive and rapid analytical method applicable for complex drug matrices.

## 2. Materials and Methods

### 2.1. Materials

High-purity flake graphite (325 meshes) and nitrogen-doped multi walled carbon nanotubes (N content: 3.00 wt %; OD: 10–20 nm; L: 0.5–2 μm; purity >95%) were purchased from Nanjing Xfnano Materials Tech. Co. Ltd. (Nanjing, China). All other reagents including urapidil, H_2_O_2_, K_4_[Fe(CN)_6_], H_2_SO_4_, and hydrazine hydrate were bought from Aladdin Chemistry Co. Ltd. (Shanghai, China). All reagents were of guaranteed grade and used as received. Double distilled water (DDW) was used for all preparations. Urapidil sustained-release tablets (Xi’an Lijun Pharmaceutical Co., Xi’an, China) were purchased from a local drugstore (Zhengzhou, China). 

### 2.2. Preparation of GO-NCNTs Dispersion

Graphene (GO) sheets were prepared from high-purity flake graphite by a modified Hummers method [28]. After preparation, GO was centrifuged at 4000 rpm to remove the unreacted flake graphite. Then, the exfoliated GO was obtained by ultrasonication for 3 h. Finally, 3 mg NCNTs were added to 10 mL 3 mg·mL^−1^ GO suspension, and then sonicated for 30 min to obtain GO-NCNTs homogeneous dispersion.

### 2.3. Preparation of 3DG-NCNTs Network Modified GC Electrode

Prior to surface functionalization, a glassy carbon (GC) electrode was polished (with aqueous slurries of 0.05 μm α-alumina on a polishing cloth) and sonicated in DDW for 5 min, after being dried under N_2_ blowing. The cleaned GC electrode was immersed in GO-NCNTs dispersion, and electrodeposition was carried out by PPM under stirring. The parameters of electrodeposition were optimized according to our previous report [29,30] and listed as follows: anodic pulse duration *t*_a_, 0.7 s; cathodic pulse duration *t*_c_, 0.3 s; experimental time *t*_exp_, 150 s. At the same time, a 3DG modified GC electrode was also fabricated by a similar procedure in GO dispersion.

### 2.4. Instruments and Characterizations

Atomic force microscopy (AFM) analysis was performed by a Dimension FastScan AFM (Bruker Company, Karlsruner, Germany). Scanning electron microscopic (SEM) images of the prepared 3DG and 3DG-NCNTs networks were obtained using a field emission scanning electron microscopic (FESEM) of MERLIN (Zeiss Company, Jena, Germany). Raman spectra were conducted on an inVia Reflex Raman spectrometer (Renishaw Company, Gloucestershire, UK). UV-vis absorption spectra were recorded on a UV-2102 spectrophotometer (UNICO Company, Shanghai, China). Electrochemical tests including cyclic voltammetry (CV), differential pulse voltammetry (DPV), chronampermetry (CA), and chronocoulometry (CC) were done on a CHI 650A electrochemical analyzer (CHI Instrumental, Shanghai, China) using a conventional three-electrode system.

## 3. Results and Discussion

### 3.1. Characterization of GO-NCNTs Dispersion

**Figure 1:**
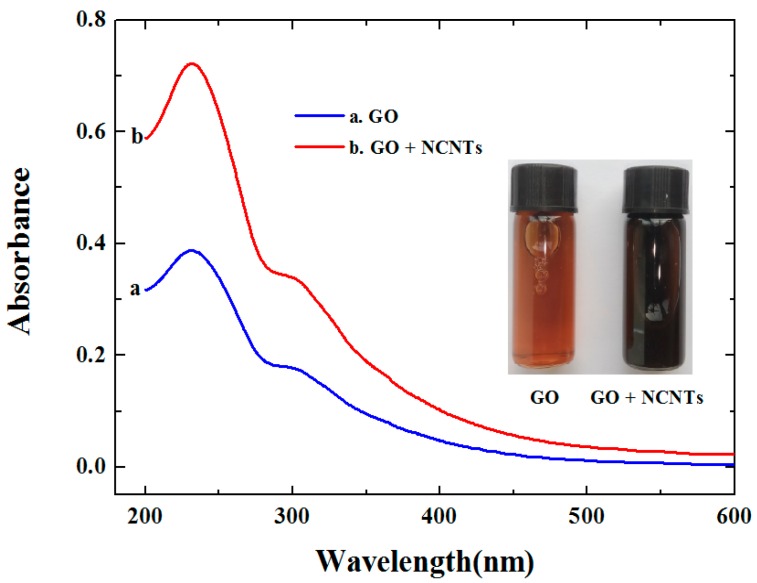
UV-visible absorption spectra of graphene-oxide (GO) (**a**) and graphene oxide-nitrogen doped carbon nanotubes (GO-NCNTs) (**b**); the inset shows the objects of GO (**a**) and GO-NCNTs (**b**).

It has been reported previously that CNTs can be bonded to GO through π-π interaction [31]. Therefore, NCNTs can also be incorporated to GO sheets. The inset of Figure 1 shows the GO (curve a) and GO-NCNTs (curve b) homogeneous dispersion. It is clear that the change of color from yellow to black was found after the NCNTs were added and dispersed. Moreover, UV-vis absorption spectra experiments were used to compare the absorption band change of GO and GO-NCNTs. As can be seen in Figure 1, the characteristic absorptions of GO are observed at about 240 nm and 310 nm, and GO-NCNTs give an obvious increase in absorbance, indicating that NCNTs have been bonded to GO sheets through π-π interaction.

**Figure 2:**
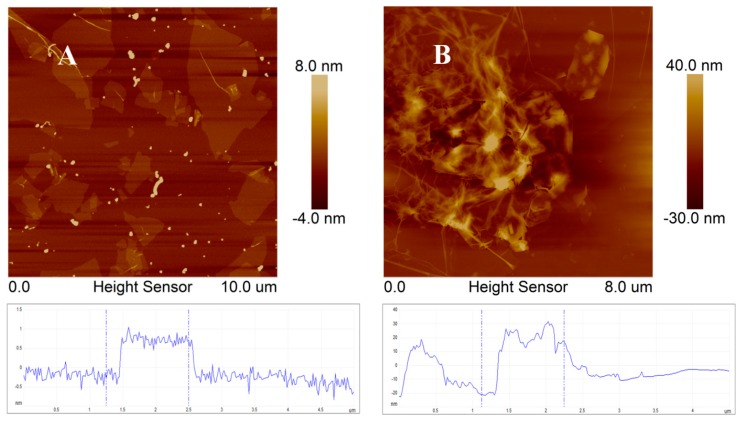
Atomic force microscopy (AFM) image of GO (**A**) and GO-NCNTs (**B**) from their dilute aqueous dispersion on freshly cleaned mica.

Further the AFM image showed the morphology of GO and GO-NCNTs (Figure 2). The result exhibited that the average thickness of GO sheets is ≈1 nm; suggesting GO sheets were almost single-layer (Figure 2A). However, the average thickness of GO-NCNTs increased to about 30 nm (Figure 2B), and many nanowires on the surface of GO sheets were observed, which further confirmed that the NCNTs have been successfully bonded onto GO.

### 3.2. Electrochemical Deposition and Characterization of 3DG-NCNTs Network

According to a previous report, the graphene sheets prepared by the CV technique were mainly laid down at the surface of the electrode (2D planar structure) [32]. On the other hand, the potentiostatic method (PM) can fabricate the 3D graphene network with a porous structure [32]. Recently, we reported that PPM can make uniform and crumpled reduced graphene sheets [29,30]. The reduced graphene sheets are nearly vertically aligned to the surface of the electrode. Therefore, we decided on PPM to fabricate the 3DG and 3DG-NCNTs modified electrode. 

**Figure 3:**
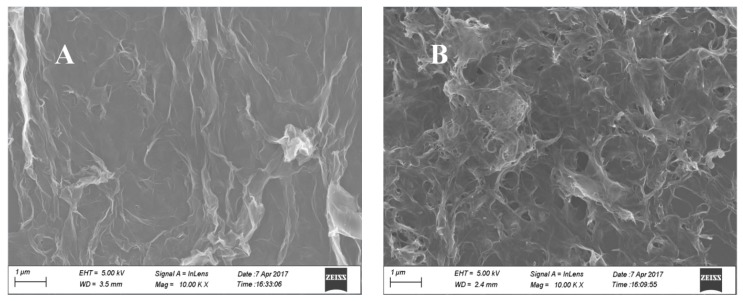
Scanning electron microscopy (SEM) images obtained from 3DG (**A**) and 3D graphene-nitrogen doped carbon nanotubes (3DG-NCNTs) (**B**) modified glassy carbon (GC) electrode.

**Figure 4:**
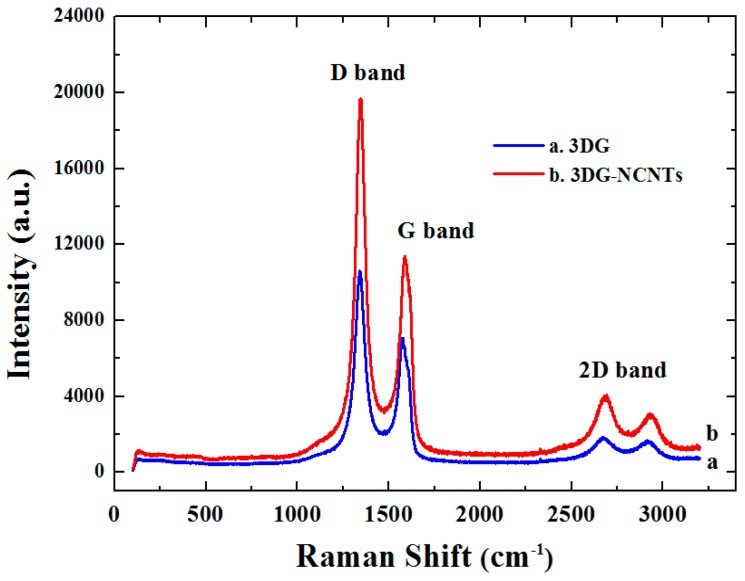
Raman spectra of 3DG (curve **a**) and 3DG-NCNTs (curve **b**) modified GC electrode.

To evaluate the structural characterization of the 3DG and 3DG-NCNTs films prepared by PPM, morphologies of the 3DG (Figure 3A) and 3DG-NCNTs (Figure 3B) modified electrodes were characterized using SEM. As can be seen in Figure 3, a highly porous and uniform interconnected network of 3DG and 3DG-NCNTs is observed on the electrode surface, respectively. The porous walls consist of upright oriented graphene sheets, which facilitate the access of target analyte to the electrode surface. Besides, to this 3DG-NCNTs network, NCNTs like a wire connect the isolated graphene sheets. The 3DG and 3DG-NCNTs network modified electrode was further characterized by Raman spectra. Figure 4 shows the Raman spectra of 3DG (curve a) and 3DG-NCNTs (curve b) modified GC electrode. The Raman spectra of 3DG and 3DG-NCNTs have two dominant peaks around 1310 and 1580 cm^−1^, which are assigned to the D and G bands of graphene sheets, respectively. From the Raman spectra of 3DG, the intensity ratio of D to G band peaks is calculated as 1.6. However, by introducing NCNTs to the 3DG, the I_D_/I_G_ ratio greatly increased to 1.8 and a distinct 2D band appeared, indicating the restoration of sp^2^ conjugated domains in the graphitic structures [33]. 

**Figure 5:**
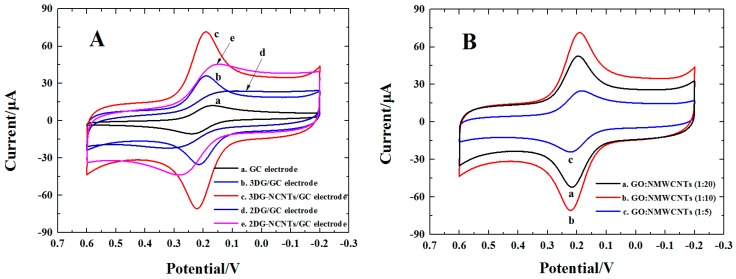
(**A**) Cyclic voltammograms (CVs) of bare GC electrode (curve a), 3DG (curve b), 3DG-NCNTs (curve c), 2DG (curve d), 2DG-NCNTs (curve e) modified GC electrode in 1.0 × 10^−3^ mol·L^−1^ K_3_[Fe(CN)_6_] + 0.1 mol·L^−1^ KCl solution; (**B**) CVs of the 3DG-NCNTs modified GC electrode prepared in different portions of NCNTs solution in 1.0 × 10^−3^ mol·L^−1^ K_3_[Fe(CN)_6_] + 0.1 mol·L^−1^ KCl solution, NCNTs portion: (a) 1:20, (b) 1:10, (c) 1:5, *v* = 0.05 V·s^−1^.

In order to demonstrate electrochemical properties, we used [Fe(CN)_6_]^3−/4−^ as a redox probe to directly investigate the charge transfer property of different electrodes [34]. Compared with the bare GC electrode, both cathodic and anodic peak currents (*i*_p_) increase significantly and peak to peak separation (Δ*E*_p_) values decrease obviously at 3DG and 3DG-NCNTs modified GC electrodes (Figure 5A), which indicate that 3DG and 3DG-NCNTs networks can offer high conductivity, respectively. Interestingly, the largest *i*_p_ and the smallest Δ*E*_p_ of the redox probe [Fe(CN)_6_]^3−/4−^ are observed at the 3DG-NCNTs modified GC electrode. The result confirms that the presence of NCNTs can increase the electron transfer at the interface and result in a more conductive network. Furthermore, the average electroactivity area of different electrodes can be obtained based on the Randles-Sevcik Equation [35]: *i*_p_ = 2.69 × 10^5^n^3/2^*AD*_o_^1/2^*c*_o_*v*^1/2^. The average electroactivity areas of the bare GC electrode, 3DG, and 3DG-NCNTs modified GC electrodes were calculated as 0.062, 0.145, and 0.232 cm^2^, respectively. The average electroactivity areas of 3DG and 3DG-NCNTs network modified GC electrodes increase significantly. This could be attributed to the formation of a highly porous 3DG and 3DG-NCNTs network. To further test that the 3D structure has a direct influence on the electrochemical performance of the modified electrode, the 3DG and 3DG-MWCNTs network modified GC electrodes were left to dry in the air. Under this circumstance, the entrapped water inside the network evaporated gradually. The result caused the 3D highly porous structure to collapse and form a compact 2D layer due to the strong π-π interactions. As a result, the *i*_p_ of the redox probe [Fe(CN)_6_]^3−/4−^ decreased dramatically and Δ*E*_p_ increase obviously in its dry state as shown in Figure 5A (curve d and curve e). Therefore, the modified electrodes must be kept in DDW to prevent the collapse of the porous network when not in use. 

The influence of the NCNTs ratio (NMWCNTs:GO) on electrochemical characterization of the 3DG-NCNTs network was also studied by using [Fe(CN)_6_]^3−/4−^ (Figure 5B). It is clear that the current grows on increasing the NCNTs. However, when the ratio of NCNTs was greater than 1:5, the current did not increase but significantly decreased and a 3DG-NCNTs film was not observed on the surface of the electrode. The reason might be that it was made difficult to electrochemically reduce the GO-NCNTs because amounts of NCNTs were bonded onto the GO. Therefore, we chose 1:10 as the optimal ratio.

### 3.3. Voltammetric Behavior of Urapidil at 3DG-NCNTs Network Modified GC Electrode

**Figure 6:**
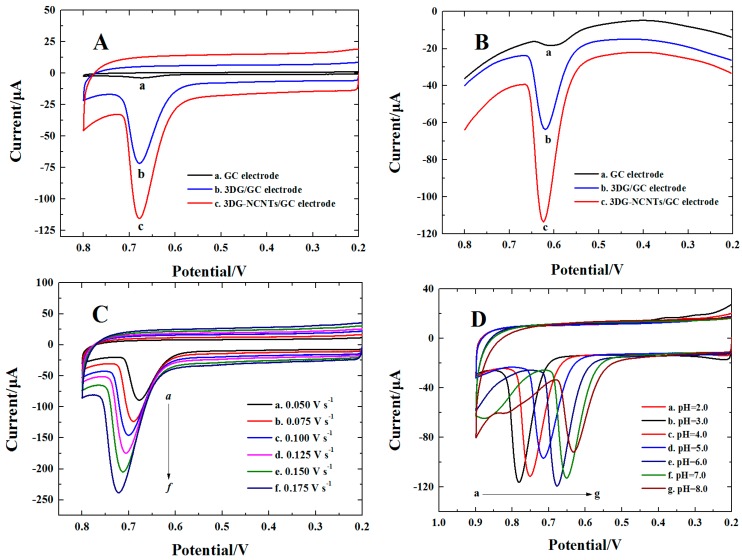
(**A**) CVs of 1.0 × 10^−6^ mol·L^−1^ urapidil on bare GC electrode (curve a), 3DG (curve b) and 3DG-NCNTs (curve c) modified GC electrode in 0.2 mol·L^−1^ phosphate buffer solutions (PBS, pH = 6.0), *v* = 0.05 V·s^−1^; (**B**) differential pulse voltammetry (DPVs) of 1.0 × 10^−6^ mol·L^−1^ urapidil on the bare GC electrode (curve a), 3DG (curve b) and 3DG-NCNTs (curve c) modified GC electrode in 0.2 mol·L^−1^ PBS (pH = 6.0); (**C**) CVs of 1.0 × 10^−6^ mol·L^−1^ urapidil on 3DG-NCNTs modified GC electrode at different scan rates; (**D**) CVs of 1.0 × 10^−6^ mol·L^−1^ urapidil on 3DG-NCNTs modified GC electrode at different pH values, *v* = 0.05 V·s^−1^.

The electrochemical response of urapidil at different electrodes was investigated by using CV and DPV techniques. Figure 6A shows the cyclic voltammetric response recorded at the bare GC electrode, 3DG and 3DG-NCNTs network modified GC electrode. As can be seen, only a very weak and broad irreversible oxidation peak is observed for urapidil on the bare GC electrode, suggesting that the oxidation activity of urapidil is very sluggish. In contrast, the oxidation peak current (*i*_pa_) increases dramatically on the surface of the 3DG and 3DG-NCNTs modified GC electrode. Also, the largest *i*_pa_ and the lowest oxidation peak potential (*E*_pa_) is obtained on the surface of 3DG-NCNTs modified GC electrode, indicating the 3DG-NCNTs network prepared is the most active for the oxidation of urapidil. The same result was also found for the electrochemical response of urapidil using the DPV technique (Figure 6B). Besides, the *i*_pa_ of urapidil on the surface of 3DG-NCNTs modified GC electrode is 20.4 and 1.9 fold higher than that of the bare GC electrode and 3DG modified GC electrode, respectively. These results can be attributed not only to the enlargement of the electroactive area of the GC electrode due to the presence of the highly porous 3DG-NCNTs network , but also to the contribution of NCNTs as electron wires that promote the conductivity of the modifier film. Therefore, the 3DG-NCNTs modified GC electrode was selected as the electrochemical sensor for investigating the voltammetric behavior of urapidil and establishing the electroanalytical method.

The kinetics for the electro-oxidation of urapidil on the surface of the 3DG-NCNTs modified GC electrode was studied by recording cyclic voltammograms (CVs) at different potential scan rates(*v*) (Figure 6C). With increasing *v*, the *i*_pa_ grows gradually and the plot of *i*_pa_ and *v* shows a good linear relationship, indicating that this is an adsorption-controlled process. Moreover, the *E*_pa_ shifts positively on increasing *v* and the plot of *E*_pa_ and ln*v* shows also a good linear relationship. The linear equation was represented as *E*_pa_ (V) = 0.770 + 0.029ln*v* (mV·s^−1^) (*R* = 0.998). According to Laviron’s theory [36], α*n* = 0.89 can be obtained from the slope. Then *n* = 2 can be achieved, with the value of α estimated from the peak width at half-height [36]. At the same time, the apparent rate constant (*k*_s_) of 1.45 s^−1^ can be also calculated from the intercept.

To illustrate the effect of pH value on the voltammetric behavior of urapidil at the 3DG-NCNTs modified GC electrode, CVs of 1.0 × 10^−6^ mol·L^−1^ urapidil were recorded at different pHs (from 2.0 to 8.0) of 0.2 mol L^−1^ phosphate buffer solutions (PBS) (Figure 6D). It was found that on increasing the pH values, the *E*_p_ shifted negatively, which suggests the participation of H^+^ ions in the oxidation reaction. Moreover, the *i*_pa_ of urapidil reached its maximum at pH = 6. Hence, PBS of pH 6.0 was used in the further experiments.

### 3.4. Chronoamperometry and Chronocoulometry Investigations

**Figure 7:**
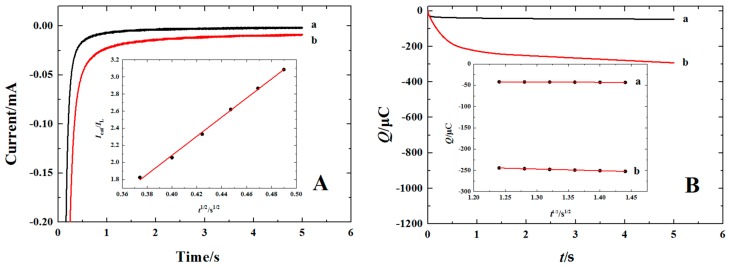
(**A**) Chronoamperometric curves of the background (curve a) and 1.0 × 10^−4^ mol·L^−1^ urapidil (curve b) in 0.2 mol·L^−1^ PBS (pH = 6.0) on the 3DG-NCNTs modified GC electrode, the inset shows the corresponding relationship of *i*_cat_/*i*_L_ versus *t*^1/2^; (**B**) Chronocoulometric curves of the background (curve a) and 5.0 × 10^−3^ mol·L^−1^ urapidil (curve b) in 0.2 mol·L^−1^ PBS (pH = 6.0) on the 3DG-NCNTs modified GC electrode; the inset shows the corresponding relationship of *Q* versus *t*^1/2^.

CA was performed to evaluate the catalytic rate constant (*k*_cat_) for the electro-oxidation of urapidil on the surface of the 3DG-NCNTs modified GC electrode. Figure 7A shows chronoamperometric curves of the 3DG-NCNTs modified GC electrode in the absence (curve a) and with 1.0 × 10^−4^ mol L^−1^urapidil (curve b). According to our previous report [30], the corresponding *i*_cat_/*i*_L_ versus *t*^1/2^ plot is presented and shown as the inset in Figure 7A. From the slope of the straight line, the *k*_cat_ is calculated to be 1.25 × 10^3^ mol L^−1^ s^−1^, which is much larger than that of the bare GC electrode (87.9 mol·L^−1^·s^−1^) and the 3DG network modified GC electrode (0.94 × 10^3^ mol·L^−1^·s^−1^). The result verifies that the 3DG-NCNTs network can provide a more efficient interface for the electro-oxidation of urapidil.

At the same time, CC was also done to determine the saturating absorption capacity (Γ^*^) for urapidil on the surface of the 3DG-NCNTs modified GC electrode. Figure 7B shows chronocoulometric curves of the 3DG-NCNTs modified GC electrode in the background (curve a) and with 5.0 × 10^−3^ mol·L^−1^ urapidil (curve b). According to the formula given by Anson [37], the corresponding *Q*~*t*^1/2^ plots are performed and shown as the inset in Figure 7B. Using the equation reported earlier: *Q* = nFAΓ^*^ [38] and the intercept difference between curves a and b, Γ^*^ is obtained to be 7.53 × 10^−9^ mol·cm^−2^. The result is much higher than that of the vbare GC electrode (9.72 × 10^−11^ mol·cm^−2^) and 3DG network modified GC electrode (2.91 × 10^−9^ mol·cm^−2^). It therefore indicates that the 3DG-MWCNTs network is effective for enlargement of the loading amount of urapidil due to enlargement of the effective surface area.

### 3.5. Analytical Application and Method Validation

#### 3.5.1. Optimization of Accumulation Conditions

**Figure 8:**
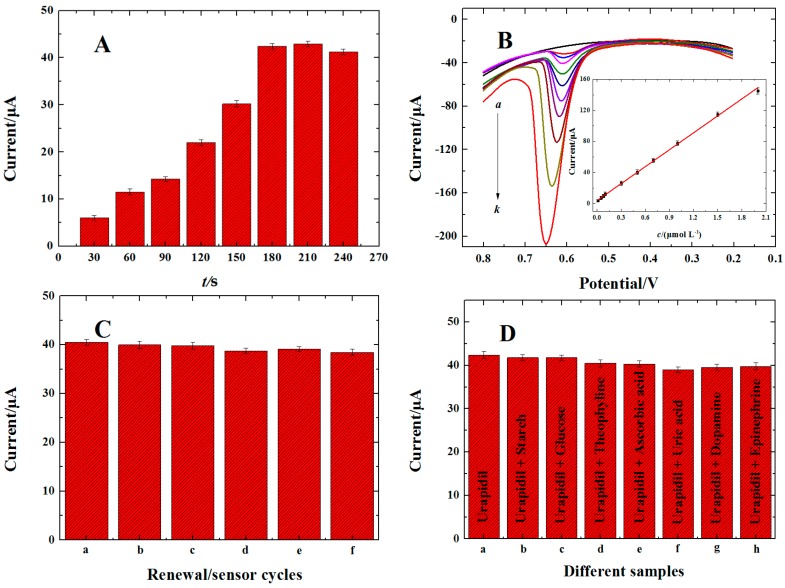
(**A**) Peak current vs. accumulation time plots for 5.0 × 10^−^^7^ mol·L^−1^ urapidil at open circuit condition; (**B**) DPASVs of different concentration of urapidil on 3DG-NCNTs modified GC electrode under the optimum conditions; the inset shows corresponding calibration curve of *c* for *i*_pa_; (**C**) Influence of renewal step on the *i*_pa_ detected on 3DG-NCNTs modified GC electrode; (**D**) Influence of different interfering compounds on the *i*_pa_ detected on 3DG-NCNTs modified GC electrode.

In the optimization of experimental parameters, the influence of the accumulation time (*t*_acc_) and accumulation potential (*E*_acc_) on the electrochemical response characteristic of urapidil was estimated by recording the *i*_pa_ using DPV. The 3DG-MWCNTs modified electrode was immersed in 5.0 × 10^−7^ mol·L^−1^ urapidil solution for a certain period of time (from 30 to 240 s) to allow the infiltration of urapidil to the inner parts of the network. As clearly shown in Figure 8A, an accumulation step at open circuit condition has a significance influence on the electrochemical response of urapidil. The *i*_pa_ increased greatly within 180 s and then enhanced only slowly on increasing *t*_acc_. As a result, 180 s was set as the accumulation time. On the other hand, the *E*_acc_(0.10–0.40 V) had little influence on the *i*_pa_. So the accumulation was carried out at open circuit condition.

#### 3.5.2. Analytical Performances

Under the optimum conditions, the calibration curve for urapidil determination was established. Figure 8B displays the response of different concentrations of urapidil by differential pulse adsorptive stripping voltammetry (DPASV). The *i*_pa_ is linearly proportional to urapidil concentrations in the range of 1.0 × 10^−8^–2.0 × 10^−6^ mol·L^−1^ as represented by the following equations: *i*_pa_ (μA) = 4.52 + 71.83*c* (μmol·L^−1^) (*R* = 0.999) (the inset of Figure 8B). A limit of detection (LOD) of 5.0 × 10^−9^ mol·L^−1^ is calculated by signal-to-noise ratio of 3 (S/N) [39]. Moreover, the proposed method is compared with previously reported methods for the electrochemical determination of urapidil [10,11] and the results are listed in Table 1. It is obvious that the proposed method exhibited a lower detection limit and wider linear ranges, which indicate that the 3DG-NCNTs networks fabricated by PPM can be used satisfactorily as electrochemical sensor materials.

The reproducibility of the modified electrode fabricated for urapidil sensing, expressed in terms of relative standard deviation, was determined to be 3.1% at a urapidil concentration of 5.0 × 10^−7^ mol·L^−1^. Renewal of the electrode could be achieved upon immersion of the electrochemical sensor in 0.2 mol·L^−1^ PBS (pH = 6.0) for 5 min under constant stirring, and CV was carried out in the potential ranging from 0.20 V to 0.80 V (vs. SCE) until the peaks of urapidil disappeared. After five renewal/sensing cycles, DPASV response to a 5.0 × 10^−7^ mol·L^−1^ urapidil solution decreased by 5.7% (Figure 8C). In addition, the long-term stability test of the sensor when stored at 4 °C for 2 weeks showed a loss of 4.2% when tested upon the addition of 5.0 × 10^−7^ mol·L^−1^ urapidil. 

#### 3.5.3. Interference Studies

To evaluate the effect of potential interfering compounds, we compared the electrochemical response toward 5.0 × 10^−7^ mol·L^−1^ urapidil before and after adding 10-fold amounts of starch, glucose, theophyline, ascorbic acid, uric acid, dopamine, and epinephrine. As seen from Figure 8D, the electrochemical response did not change significantly (≤5%). Moreover, it was also observed that 200-fold of Ca^2+^, Mg^2+^, Zn^2+^, Cu^2+^, and Fe^2+^ had almost no influence on the oxidation peak of urapidil. These results clearly confirmed that the electrochemical sensor had excellent selectivity.

### 3.6. Determination of Urapidil in a Tablet Sample

To test the feasibility of the developed method for the analysis of tablet samples, the 3DG-NCNTs modified GC electrode was applied for the determination of urapidil content in commercial tablets using a standard addition method. The tablet samples were processed according to previous work [11]. The results of the proposed method were compared to those using UV-visible methods (Table 2). The results of these tests revealed that the method is well adapted for urapidil detection in tablet samples. Compared to the UV-visible method, no significant difference was obtained.

## 4. Conclusions

In this work, we presented a simple yet effective way to fabricate 3DG-NCNTs network on the surface of a GC electrode using PPM in a GO-NCNTs homogeneous dispersion. The prepared network exhibits a larger accessible surface area as well as higher conductivity by incorporation of NCNTs into the 3D network. The 3DG-NCNTs network was applied as a new voltammetric material for the fabrication of the electrochemical platform for determination of urapidil, which showed a wider linear range and a low detection limit. Moreover, good sensitivity and selectivity make it very suitable for determination of urapidil content in tablet samples with excellent recoveries as confirmed by the UV-visible method.

## Figures and Tables

**Table 1:** Comparison of determination of urapidil by the different sensing methods reported.

Electrode	Methods	Linear Range (mol·L^−1^)	Detection Limit (mol·L^−1^)	Reference
Multi-wall carbon nanotube paste electrode	DPV	5.0 × 10^−8^~2.0 × 10^−6^	3.8 × 10^−8^	[10]
Electrochemically pretreated GC electrode	LSV	2.0 × 10^−8^~5.0 × 10^−6^	2.0 × 10^−8^	[11]
3DG-NCNTs modified GC electrode	DPV	1.0 × 10^−8^~2.0 × 10^−6^	5.0 × 10^−9^	This work

DPV: differential pulse voltammetry; GC: glassy carbon; 3DG-NCNTs: 3D graphene-nitrogen doped carbon nanotubes LSV: linear sweep voltammetry.

**Table 2:** Determination of results of urapidil in tablet samples by differential pulse adsorptive stripping voltammetry (DPASV).

DPASV (n = 3) ^a^						UV-Vis	
Samples	Original Found (μmol·L^−1^)	Standard Added (μmol·L^−1^)	Total Found (μmol·L^−1^)	RSD (%)	Recovery (%)	Original Found (μmol·L^−1^)	RSD (%)
Tablet ^b^	0.49(30.4 mg·tablet^−1^)	0.30	0.78	3.4	96.7	0.50	1.9
		0.50	1.01	2.9	104.0		
		1.00	1.45	4.1	96.0		

^a^. Average of three determinations; ^b^. Marked urapidil content: 30 mg·tablet^−1^.
